# Action Observation in People with Parkinson’s Disease. A Motor–Cognitive Combined Approach for Motor Rehabilitation. A Preliminary Report

**DOI:** 10.3390/diseases6030058

**Published:** 2018-07-04

**Authors:** Walter Di Iorio, Alessandro Ciarimboli, Giorgio Ferriero, Michele Feleppa, Luigi Baratto, Giuseppe Matarazzo, Giovanni Gentile, Stefano Masiero, Patrizio Sale

**Affiliations:** 1 Rehabilitation Unit, Villa Margherita, 82100 Benevento, Italy; Walter.diiorio@alice.it (W.D.I.); aciarimboli@libero.it (A.C.); luigi.baratto@gmail.com (L.B.); info.bn@casadicuravillamargherita.it (G.M.); 2 Division of Physical Medicine and Rehabilitation, Salvatore Maugeri Foundation, Scientific Institute of Lissone, IRCCS, 20851 Lissone, Italy; giorgio.ferriero@icsmaugeri.it; 3 Neurological Unit and Stroke Unit, Ospedale Civile, 82100 Benevento, Italy; michele.feleppa@ao-rummo.it; 4 San Camillo Hospital IRCCS, 30126 Venice, Italy; giovanni.gentile@ospedalesancamillo.net; 5 Department of Neuroscience, University of Padua, 35100 Padua, Italy; stef.masiero@unipd.it

**Keywords:** Parkinson’s disease, Action Observation, mirror neuron systems, balance, gait, rehabilitation, neural plasticity, electroencephalogram, P300

## Abstract

The aim of this study was to assess the role of Action Observation (AO) to improve balance, gait, reduce falls, and to investigate the changes in P300 pattern. Five cognitively intact People with Parkinson’s disease (PwP) were enrolled in this prospective, quasi-experimental study to undergo a rehabilitation program of AO for gait and balance recovery of 60 min, three times a week for four weeks. The statistical analysis showed significant improvements for Unified Parkinson’s Disease Rating Scale (UPDRS) motor section III *p* = 0.0082, Short form 12-items Healthy Survey (SF-12) Mental Composite Score (MCS) *p* = 0.0007, Freezing of gait Questionnaire (FOG-Q) *p* = 0.0030, The 39-items Parkinson’s Disease Questionnaire (PDQ-39) *p* = 0.100, and for P300ld *p* = 0.0077. In conclusion, AO reveals to be a safe and feasible paradigm of rehabilitative exercise in cognitively preserved PwP.

Together with pharmacological treatment, intensive physical therapy is used to improve motor performances in Parkinon’s disease (PD) patients [1]. Unfortunately, patients with poor motor ability cannot easily take part in physical therapy programs due to motor or cognitive impairment. Therefore, experience-dependent neural plasticity often proves to be a hard task for neurorehabilitation, in means of repair and recovery. However, the systematic observation of significant motor actions closely linked to their execution (action observation treatment (AOT)) has been proposed to be a valuable and feasible practice for motor-impaired patients [2]. As reported by Abruzzese and colleagues, Action Observation (AO) therapy shows its effectiveness in learning or enhancing the quality of execution of specific motor skills [3], and it has been described as an effective cognitive tool for rehabilitation [2,4,5,6], since it can shape neural circuit reorganization and promote neural plasticity and motor learning [7,8,9,10,11]. Despite this promising evidence, very few studies have been published about the rehabilitation of People with Parkinson’s (PwP) using the AO paradigm [3].

Neural activity, reflecting the cognitive load, has usually been measured through electroencephalography recording, specifically by analyzing the event-related potentials (ERPs), which show the synchronous activity of large groups of neurons firing together [12]. Among ERP components, the late positive wave deflection component, known as P300, has been identified as an element reflecting cognitive processes related to sustained and focused attention, to short-, long-term, and working memory, stimulus detection, and decision making tasks [13,14,15,16].

The specific aim of this short communication is to assess the role of Action Observation as a rehabilitative tool for improving balance, gait, reducing falls, and to investigate the changes in P300 pattern duo to the treatment effects.

Five cognitive intact people with Parkinson’s disease (PwP) were enrolled and assigned the treatment. Each subject was informed about the study procedure and aims and then, after a period of discussion and reflection either enrolled voluntarily and provided written informed consent or declined to participate. All procedures conformed to the World Medical Association declaration of Helsinki. PwP were considered eligible and enrolled, based on the following criteria: Diagnosis of idiopathic PD according to UK Brain Bank criteria; age between 18–80; ability to walk with minimal assistance for 25 feet; ability to stand for 20 min without support; stable Parkinson’s medication treatment over the last 4 weeks preceding the enrolment; Mini-Mental State Examination >25/30; HAM-D (Hamilton Depression Scale) <17. The following exclusion criteria were considered: Other significant neurologic, cardiac, or orthopedic comorbidities; and chronic abuse of alcohol.

The clinical assessment was performed by blind judges at baseline (T_0_) and immediately after the end of the treatment (T_1_). Judges were trained professionals, not involved in the rehabilitation program. All the clinical outcomes were collected during the ON state, one hour and a half after the last intake of the usual dose of levodopa. Both assessments were done at baseline (T_0_) and at the end of the rehabilitation program (after 12 AO sessions) (T_1_), or no later than one day after the last training session. The clinical and quantitative outcomes were collected by using the standard instruments for clinical assessment of PwP: Hoehn & Yahr Scale; Unified Parkinson’s Disease Rating Scale (UPDRS) motor section III, Mini Mental State Examination (MMSE), Freezing of gait Questionnaire (FOG-Q), Timed Up & Go Test (TUG), Ten meters walking test (steps and seconds) (10 MWT), Berg Balance Scale (BBS), The 39-items Parkinson’s Disease Questionnaire (PDQ-39), Short form 12-items Healthy Survey (SF-12) with both Physical and Mental Composite Score (PCS, MCS), and Functional Independence Measure (FIM). Recording of ERPs and P300 extraction was performed in agreement with standard procedures [17], by presenting to the subjects, a series of acoustic stimuli divided into two different classes of target and non-target stimuli. Target stimuli were programmed to be less frequent than non-target stimuli. Our subjects performed this procedure before and after the rehabilitative treatment (T_0_ and T_1_).

All participants underwent two 30-min daily sessions of the assigned treatment. Tasks for the reduction of freezing of gait aspects were based on strategies like tapping the ankle, taking lateral or posterior steps, and counting aloud while walking. During each daily session, the patient was asked to watch one single action presented by the physical therapist and to get ready to imitate the presented action. Each subject was also asked to observe, by paying attention, to the lower limb goal-oriented movement of the therapist sitting in front of him (the therapist’s left hand was located just in front of the patient’s right hand), without producing any movement, nor imagining any movement.

The single actions were:

1. Rising from a chair without the help of the upper limbs.
2. Alternating monopodial support for 2–3 s per side.
3. Going through a parallel walking bar (approximately 3 m) and overriding a small wood obstacle, about 15 cm in height.
4. Going out from parallel walking bars.
5. Change of direction while walking: 90° turn to the right.
6. Change of direction while walking: 90° turn to the left.
7. Walking through a narrow space consisting of two beds closely displaced (about 2 m).
8. Going back to the starting point (chair).

At the end of each sequence, the therapist encouraged the patient to perform the observed motor action movement over the subsequent 15 min, providing help as needed. The observed action was performed, indicating to patients to repeat it as many times as they could. Each session was repeated twice per day in two separate sessions, at least 60 min apart. All actions were object and goal-directed. The therapist maintained the patient’s attention with verbal feedback (Figure 1). 

A preliminary descriptive analysis was performed to check the normal distribution of patients’ clinical and instrumental data using Shapiro-Wilk test. Whereby the variables collected presented a normal distribution, parametric statistic tests were used. Non-parametric tests were instead used where the normality distribution criteria wasn’t satisfied. Specifically, the Wilcoxon signed-ranks test was performed to determine the location of any significant differences between time points. The alpha level for significance were set at *p* < 0.05 for the first level of analysis. 

The distribution of the study sample (*n* = 5) by age and main clinical and demographical characteristics are reported in Table 1. The study didn’t have any dropouts during the treatment and all subjects enrolled were able to complete the program. Moreover, no issues have been registered during the execution of the AO program, since every precaution required in a structured rehabilitation facility was taken. In Table 1, the descriptions of all the outcome variables are presented, both measured at baseline T0 (N = 5) and post-treatment T1 (N = 5) (Table 1). The Wilcoxon test analysis showed statistically significant improvements for UPDRS motor section III *p* = 0.0082, SF-12 MCS *p* = 0.0007, FOG-Q *p* = 0.0030, PDQ-39 *p* = 0.100, and for P300LD *p* = 0.0077, results are also shown in Table 1. No statistically significant improvements for other scales were found. Furthermore, a correlation analysis using Spearman’s *r* was performed in order to enlighten the exact role of the P300 change over clinical outcomes. The results did show strong coefficient value of Spearman *r* between Δ_T1-T0_ change of clinical outcomes (BBS, SFM-12 MCS, PDQ-39, and FIM scores), and the latency duration Δ_T1-T0_ change of P300. Despite the high strength value of correlation, no statistical significance was found for any of the reported outcomes, primarily due to the limited sample size included in this study.

In the present paper, a non-invasive rehabilitation approach based on selected observation of action and imitation has been proposed in order to improve motor and cognitive performance [18]. To our knowledge, so far only two studies have shown a positive effect of Action Observation Therapy in Parkinson disease. In the first study, Buccino and co-workers have demonstrated that these original approaches, in addition to conventional rehabilitation, can significantly improve dexterity and independence in daily activities in PD [19]. In particular, the study investigated the effectiveness of rehabilitative treatment with AO in 15 subjects with PD. Individuals improved significantly more than controls in the UPDRS score and the Functional Independence Measure (FIM) scale [3,19]. In the second study, Pelosin and colleagues have shown positive additional effects on walking ability recovery and freezing of gait reduction; in particular, the authors investigated whether AO, combined with practicing the observed actions, was able to reduce the frequency Freezing of Gait (FOG) episodes in PD [20]. Our results are in accordance with Pelosin and colleagues about the recovery of motor performances (UPDRS III), SF-12 MCS, FOG-Q, and PDQ-39 [3,20]. The novelty of our protocol is related to the introduction of P300 assessment for the detection of changes in brain activity of subjects attending an AO program. As Abruzzese and colleagues highlight, it is widely accepted that the observation of actions performed by others can provoke in the brain the same activation of neural structures recruited for the actual execution of those actions [3]. Specifically, Buccino and colleagues say that Action Observation therapies are founded on the principles of movement “imitation”, implying at the same time motor imagery, observation, and actual movement execution [3]. Previous studies have shown a positive correlation between the prolongation of P300 latencies in PD and cognitive deficits in memory, attention, executive function domains, and depression [21,22,23,24]. Moreover, prolonged P300 latencies are correlated with cognitive impairment and wider behavioral problems in PD patients [25]. Our results showed an improvement in cognitive and motor performances that seems to be related with a reduction of P300 LD signals by means of clinical scales score, related to motor, cognitive, and quality of life assessment, according to current literature [26,27,28,29,30,31]. In conclusion, AO appears to be a safe and feasible paradigm of rehabilitative exercise in cognitively preserved PwP. Moreover, our study suggests that AO practice produces a combined effect both on walking skills recovery and cortical activity changes in PwP. This approach could contribute to increase lower limb motor recovery in idiopathic PD patients. The results of this study, despite the small sample size of subjects enrolled and treated with AO, were also encouraging and supported by recent research works in Parkinson’s disease cognitive and motor rehabilitation [18,32]. In general, the simplicity of treatment, the lack of side effects, and the positive results from patients support our theory. Although, AO exercises and training effects over gait and cognitive recovery need to be further investigated. Future research should include a larger sample size and analyze in depth the underlying central and peripheral neural mechanisms over time using long-term follow-up.

## Figures and Tables

**Figure 1:**
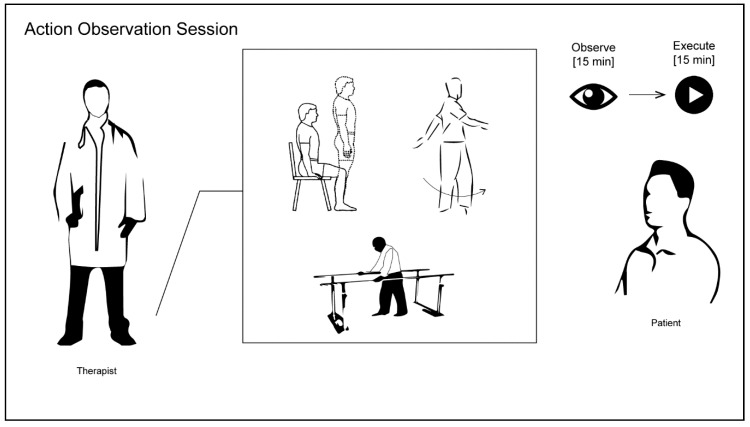
Overview of Action Observation session: The patient first observes the single action (e.g., rising from a chair, 90° walking turn, going through walking parallel bars) performed by the physical therapist, then is asked to execute the action previously observed.

**Table 1:** List of outcomes measures and clinical scales administered pre and post treatment. H&Y: Hoehn & Yahr Scale; UPDRS III: Unified Parkinson’s Disease Rating Scale motor section III; MMSE: Mini Mental State Examination; FOG-Q: Freezing of gait Questionnaire; Time U&G: Timed Up & Go Test; 10 m Walk Test: Ten meters walking test (steps and seconds); BBS: Berg Balance Scale; PDQ-39: The 39-items Parkinson’s Disease Questionnaire; SF-12: Short form 12-items Healthy Survey Physical and Mental Composite Score (PCS, MCS); FIM: Functional Independence Measure; P300 Latency Duration (LD); T_0_ vs. T_1_ (*p*): Shapiro-Wilk test; *r*: Spearman’s *r* correlation test between Δ_T1-T0_ changes of each clinical measure with P300 latency duration .

		Mean	Std. Deviation	Std. Error of Mean	T_0_ vs. T_1_	*r*	25% Percentile	Median	75% Percentile
Ages of diseases		8	4.528	2.025			4.5	6	12.5
Gender	5 Male	0 female							
Age		71.6	6.731	3.01			66	74	76
UPDRS III	T0	34.6	12.82	5.732			23.5	33	46.5
UPDRS III	T1	24.2	13.33	5.962	*p* = 0.0082	−0.102	13	22	36.5
Hoeh&Yahr	T0						2.5	2.5	2.5
Hoeh&Yahr	T1				n.s.	−0.288	2	2.5	2.5
BBS	T0	45.4	10.45	4.675			37	50	51.5
BBS	T1	52.8	2.95	1.319	n.s.	0.790	50.5	53	55
10 m walk Sec	T0	13.36	6.628	2.964			7.4	11.9	20.05
10 m walk Sec	T1	10.41	2.258	1.01	n.s.	0.200	8.35	9.95	12.7
10 m walk Step	T0	20.8	4.266	1.908			17	20	25
10 m walk Step	T1	20.6	2.881	1.288	n.s.	−0.011	18.5	20	23
Time U&G	T0	17.96	11.41	5.101			10.35	15.3	26.9
Time U&G	T1	12.62	3.259	1.458	n.s.	0.300	9.7	12.4	15.65
SF12 PCS	T0	33.12	2.758	1.233			30.65	32.6	35.85
SF12 PCS	T1	41.28	9.055	4.05	n.s.	−0.300	33.4	45.1	47.25
SF12 MCS	T0	37.86	6.433	2.877			31.85	40.8	42.4
SF12 MCS	T1	49.9	6.6	2.952	*p* = 0.0007	−0.900	44	51.2	55.15
FOG-Q	T0	15	1.871	0.8367			13.5	15	16.5
FOG-Q	T1	8.4	2.302	1.03	*p* = 0.003	0.300	6.5	8	10.5
PDQ-39	T0	60.4	34.54	15.45			32	49	94.5
PDQ-39	T1	36	23.87	10.68	*p* = 0.01	−0.900	16	27	60.5
FIM	T0	92.6	8.792	3.932			84.5	92	101
FIM	T1	103.2	10.52	4.705	n.s.	0.700	93.5	108	110.5
MMSE	T0	25.71	1.63	0.7292			24.37	24.97	27.43
MMSE	T1	25	0.9925	0.4438	n.s.	−0.300	24	25.3	25.85
P300 LD	T0	379.5	42.08	21.04					
P300 LD	T1	349.8	33.01	16.50	*p* = 0.0077				
